# Complementing Cancer Metastasis

**DOI:** 10.3389/fimmu.2018.01629

**Published:** 2018-07-16

**Authors:** Dawn M. Kochanek, Shanawaz M. Ghouse, Magdalena M. Karbowniczek, Maciej M. Markiewski

**Affiliations:** Department of Immunotherapeutics and Biotechnology, School of Pharmacy, Texas Tech University Health Sciences Center, Abilene, TX, United States

**Keywords:** complement system proteins, cancer, metastasis, alveolar macrophages, myeloid-derived suppressor cells, epithelial–mesenchymal transition

## Abstract

Complement is an effector of innate immunity and a bridge connecting innate immunity and subsequent adaptive immune responses. It is essential for protection against infections and for orchestrating inflammatory responses. Recent studies have also demonstrated contribution of the complement system to several homeostatic processes that are traditionally not considered to be involved in immunity. Thus, complement regulates homeostasis and immunity. However, dysregulation of this system contributes to several pathologies including inflammatory and autoimmune diseases. Unexpectedly, studies of the last decade have also revealed that complement promotes cancer progression. Since the initial discovery of tumor promoting role of complement, numerous preclinical and clinical studies demonstrated contribution of several complement components to regulation of tumor growth through their direct interactions with the corresponding receptors on tumor cells or through suppression of antitumor immunity. Most of this work, however, focused on a role of complement in regulating growth of primary tumors. Only recently, a few studies showed that complement promotes cancer metastasis through its contribution to epithelial-to-mesenchymal transition and the premetastatic niche. This latter work has shown that complement activation and generation of complement effectors including C5a occur in organs that are target for metastasis prior to arrival of the very first tumor cells. C5a through its interactions with C5a receptor 1 inhibits antitumor immunity by activating and recruiting immunosuppressive cells from the bone marrow to the premetastatic niche and by regulating function and self-renewal of pulmonary tissue-resident alveolar macrophages. These new advancements provide additional evidence for multifaceted functions of complement in cancer.

## Introduction

In both mouse models of cancer and patients, the expression of several complement genes is increased, resulting in higher than normal concentrations of complement proteins in plasma or other body fluids (1, 2, 3). In addition, complement activation is thought to occur in cancers because activated complement fragments are deposited within tumors (4, 5). This deposition of complement cleavage products and complement protein complexes including the C5b-9 terminal complement complex was observed in breast cancer (6) and in papillary thyroid carcinoma (7, 8). Complement activation through the lectin pathway was shown in colorectal carcinoma (9, 10). Complement fragments were detected in ascites from ovarian carcinoma patients (11). Complement activation in cancer patients is also supported by detection of C5a circulating in plasma of non-small cell lung carcinoma patients (2). The early studies reporting upregulation and activation of the complement pathway led to a notion that complement, similar to lysing bacteria, may contribute to lysis of tumor cells and, consequently, participates in tumor immune surveillance. However, this is disputable because of the resistance of cancer cells to complement-mediated lysis, which, however, become obvious mainly in the context of use of monoclonal antibodies for cancer immunotherapy (12, 13). This resistance results from high expression of membrane complement regulatory proteins (CRPs) on tumor cells (14) and secretion of soluble complement regulators from these cells (15), especially in solid tumors (13, 16). In contrast, in hematologic malignancies, complement mediated killing can be relevant, at least in the therapeutic context. For example, rituximab, a chimeric CD20 monoclonal antibody used to treat B cell lymphomas utilizes complement-mediated cytotoxicity (CDC) to kill tumor cells. There is growing interest in targeting complement regulators to improve efficacy of monoclonal antibody therapy in cancer (13, 17). Another approach to improve complement-mediated killing of tumor cells is the use of the “hexabody” platform. This technology stems from a seminal discovery that IgGs form hexamers after binding to antigen on the activating surface. This process is mediated by noncovalent interactions between Fc fragments of IgGs (18). Engineering Fc segments can be utilized to enhance formation of hexamers and, consequently, improvement of CDC toward tumor cells (19). Additional example of antitumor complement functions is participation of the complement anaphylatoxins C3a and C5a in enhancing antitumor immunity after radiotherapy. Interestingly, dexamethasone, a drug often administrated during radiotherapy limited complement activation and, consequently, inhibited antitumor immunity (20).

In contrast to these beneficial outcomes of complement activity, it is conceivable that without the discussed here therapeutic interventions, complement enhances tumor growth through its proinflammatory properties (4, 5). This possibility is consistent with a well-established tumor promoting role of chronic inflammation (21). Indeed, the first work to demonstrate tumor promoting properties of complement showed that several complement deficiencies were associated with reduced tumor growth through mechanisms linked to improvement of antitumor immunity (22). Several follow-up studies demonstrated immunoregulatory properties of various complement proteins (23). In addition, complement enhances tumor growth through direct regulation of tumor cell proliferation and invasiveness through C3a and C5a receptors expressed on carcinoma cells (24). Interestingly, the receptors for anaphylatoxins are also expressed in several leukemia and lymphoma cell lines and the blasts from chronic myeloid leukemia and acute myeloid leukemia patients. These cells responded robustly to C3a and C5a stimulation *in vitro* through chemotaxis and this process is negatively regulated by heme oxygenase 1 (HO-1) (25). These findings indicate that trafficking and spread of tumor cells in hematologic malignancies is perhaps, at least partially, controlled by complement system, therefore, inhibiting complement or upregulating HO-1 offer a new therapeutic opportunity for hematologic malignancies.

Together, studies of the last decade provide compelling evidence for a pivotal role of the complement system in tumor growth and targeting complement for anticancer therapy. Interestingly, recent developments point to regulation of cancer metastasis by complement, which appears, in some studies, to be independent from complement functions in primary tumors. This work links complement to a phase of metastatic process that only recently has been proved experimentally and is termed the premetastatic niche (26). We focus our discussion here on these new advancements on complement in metastasis. We also discuss contributions of complement to epithelial-to-mesenchymal transition (EMT), which initiates metastasis in primary tumors.

## Complex Complement

The complement system is an assembly of more than 50 proteins that work together to provide immunity from infections, regulate several homeostatic processes, and trigger responses to tissue damage or injury (23). Although the textbook definition places complement in the center of innate immunity, recent developments demonstrated that this versatile system functions beyond limits of the immune system, regulating, for example, synaptic pruning (27), tissue regeneration/repair (28, 29), and bone homeostasis (30). In addition to its key function in innate immunity, complement regulates adaptive immunity. The receptors for the complement activation fragments are expressed in B and T cells and their signaling is pivotal for maintaining efficient protection against infection (31, 32). The stimulation of the complement receptor 2 (CR2) through antigen coated with C3d reduces the threshold for B cell activation rendering costimulation for best antibody production (33, 34). The studies on a role of complement in regulating T cell responses has led to surprising discovery that complement proteins in the cytoplasm regulate several intracellular process, mainly of a metabolic nature, essential for T cell homeostasis. The intracellular complement, termed “complosome,” interacts with other intracellular innate sensor systems to control processes that are fundamental for adaptive immune responses such as metabolic reprograming necessary for generation of effector T cells (35).

The complement system also includes soluble fluid-phase or membrane-bound proteins, cofactors, regulators, and receptors (36). Upon stimulation by either pathogen or danger-associated molecular patterns, or antibodies, a cascade of events occurs that leads to activation of complement through different complement pathways. The alternative pathway is initiated by bacterial surfaces or unconstrained fluid phase hydrolysis of the complement C3 thioester (37). The lectin pathway is triggered through binding of mannose binding lectin or the ficolins (termed ficolin-1, ficolin-2, ficolin-3) to particular carbohydrates or N-acteyl residues (38, 39). The classical pathway starts when C1q binds to at least two IgG molecules (or one IgM) in a complex with antigen (40). In addition, complement fragments can be cleaved and thus activated through proteolytic enzymes that are not traditionally linked to the complement system. We grouped these additional ways of complement activation under the “umbrella” of the “fourth extrinsic pathway” (41). All three traditional complement activation pathways lead to cleavage of a complement fragment C3, which results in generation of C3a anaphylatoxin (10 kDa) and a large component—C3b. The C3b is deposited on the bacterial or other activating surfaces (42, 43). Following cleavage of C3 by an enzymatic complex—C3 convertase, C5 is cleaved by C5 convertase and similar to C3 cleavage, small C5a and large C5b fragments are generated. C3b and C4b opsonize pathogens, e.g., flag them for phagocytosis by myeloid professional phagocytes that express receptors for C3 cleavage fragments. The large C5 cleavage product, C5b, binds to an activating surface and supports subsequent binding of C6, C7, C8, and finally C9 [membrane attack complex (MAC)]. The multiple C9 fragments polymerize and form a pore in the cell membrane resulting in cell lysis or cell activation in certain circumstances (44). The complement anaphylatoxins C3a, C4a, and C5a are potent mediators that orchestrate events of inflammation (41, 45).

Excessive complement activation can be deleterious; therefore, this process is tightly controlled by CRPs (46). There are both soluble and membrane bound CRPs that can be grouped into several functional categories: (i) CRPs with decay-acceleration activity that increases the rate of C3 convertase breakdown and (ii) with cofactor activity resulting in the cleavage of C3b and C4b, thus, stopping C3 convertase formation (47). Three additional important CRPs are factor H, C1 inhibitor (C1NH), and CD59. Factor H acts in the alternative pathway as a C3 convertase decay accelerator and as a cofactor for factor I-mediated cleavage of C3b. CD59 is the only CRP, which acts to prevent assembly of the MAC. C1NH acts in both the classical and lectin pathways by inactivating C1r, C1s, and mannose-binding lectin serine proteases (47, 48, 49).

## Complement and Cancer

In 2008, complement C3, C4, and C5a receptor 1-deficient mice were shown to have slower tumor growth in a model of human papilloma virus-induced cancer (22). This paper was the first study to contradict a well-accepted, at that time, notion of complement participation in immune surveillance. Tumor promoting functions of complement, at least in this model, were linked to C5a/C5a receptor 1 (C5aR1)-mediated activation and recruitment of myeloid-derived suppressor cells (MDSC) to tumors and inhibition of antitumor immunity. At the time of this publication, concerns were raised that the observed phenotypes may be restricted to a single tumor model (4, 5, 50). However, multiple preclinical and clinical studies in the last decade supported tumor-promoting properties of different complement components (16, 49). For example, studies by Corrales and colleagues demonstrated that C5a regulates MDSC in a lung cancer model (2). The blockade of C5aR1 led to a reduction in expression of genes that suppress antitumor immunity including *Arg1, Il-6, Il-10, Ctla4, Lag3, and Cd234 (PDL1)* (2). Recently, it has been shown that C3aR and C5aR1 signaling have an important impact on the IL-10-mediated cytotoxic properties of CD8^+^ T cells infiltrating tumors in models of melanoma and breast cancer (E0771) (51). In this manuscript, tumor infiltrating CD8^+^ T cells were shown to produce C3, which in autocrine manner inhibited the expression of IL-10. This cytokine appears to be essential for the cytotoxic properties of these cells. Mechanistically, IL-10 was associated with C3aR and C5aR1 signaling in CD8^+^ T cells. Complement’s role in recruiting tumor-associated macrophages (TAMs) and controlling their proangiogenic characteristics was proposed in a work, exploring antitumor functions of pentraxin 3 (52).

In addition to research demonstrating contributions of complement to inhibition of antitumor immunity, several studies showed other mechanisms behind tumor promoting functions of complement. In ovarian carcinoma models, tumor cells were demonstrated to produce complement components. C3a and C5a generated through activation of complement fragments produced in tumor cells regulated proliferation and invasiveness of tumor cells in autocrine fashion (24). C1q deposited in several human malignancies and mouse tumors seems to accelerate tumor growth through its proangiogenic properties and direct regulation of tumor cell motility and proliferation (53). Of value, Ajona et. al recently demonstrated improved efficacy of programmed cell-death 1 (PD-1) blockade in the presence of complement inhibition in reducing progression of tumors in a model of lung cancer (54). These new findings divulge a feasible path for targeting the complement system with the use of immunotherapeutic agents along with T cell check inhibitors. The detailed and comprehensive descriptions of a role in regulating tumor growth can be found in recent reviews (16, 23, 49). Here, we focus the discussion on the role of complement in regulating metastasis, a role that seems to involve different mechanisms.

## Metastasis a Hallmark of Malignancy

Cancer metastasis is a process of relocation of tumor cells from a primary to a distant (disconnected from primary tumor) site, through lymph or blood. In fact, a metastatic potential determines the malignant character of primary growth (55). Cancer metastasis are responsible for approximately 90 percent of cancer-associated deaths, however, paradoxically, mechanisms regulating metastasis remain the most obscure aspect of cancer biology (56). The metastatic spread of cancer is a multistep and complex chain of alterations in tumor and host cells, and tumor stroma, known as the invasion-metastasis cascade (56, 57). This cascade involves processes in primary tumor sites, circulation, and metastasis-targeted organs. Some of the first steps in the metastatic cascade involve acquisition of the ability to migrate and invade and degrade the tumor stroma by tumor cells. This goal is achieved through triggering in tumor cells several cellular programs that are collectively termed EMT, which is also an essential process during embryogenesis and wound healing (58). The EMT occurs perhaps in several malignancies; however, the current understanding of these cellular adaptations stems from studies in the models of epithelial-origin neoplasms carcinomas (59).

**Figure 1:**
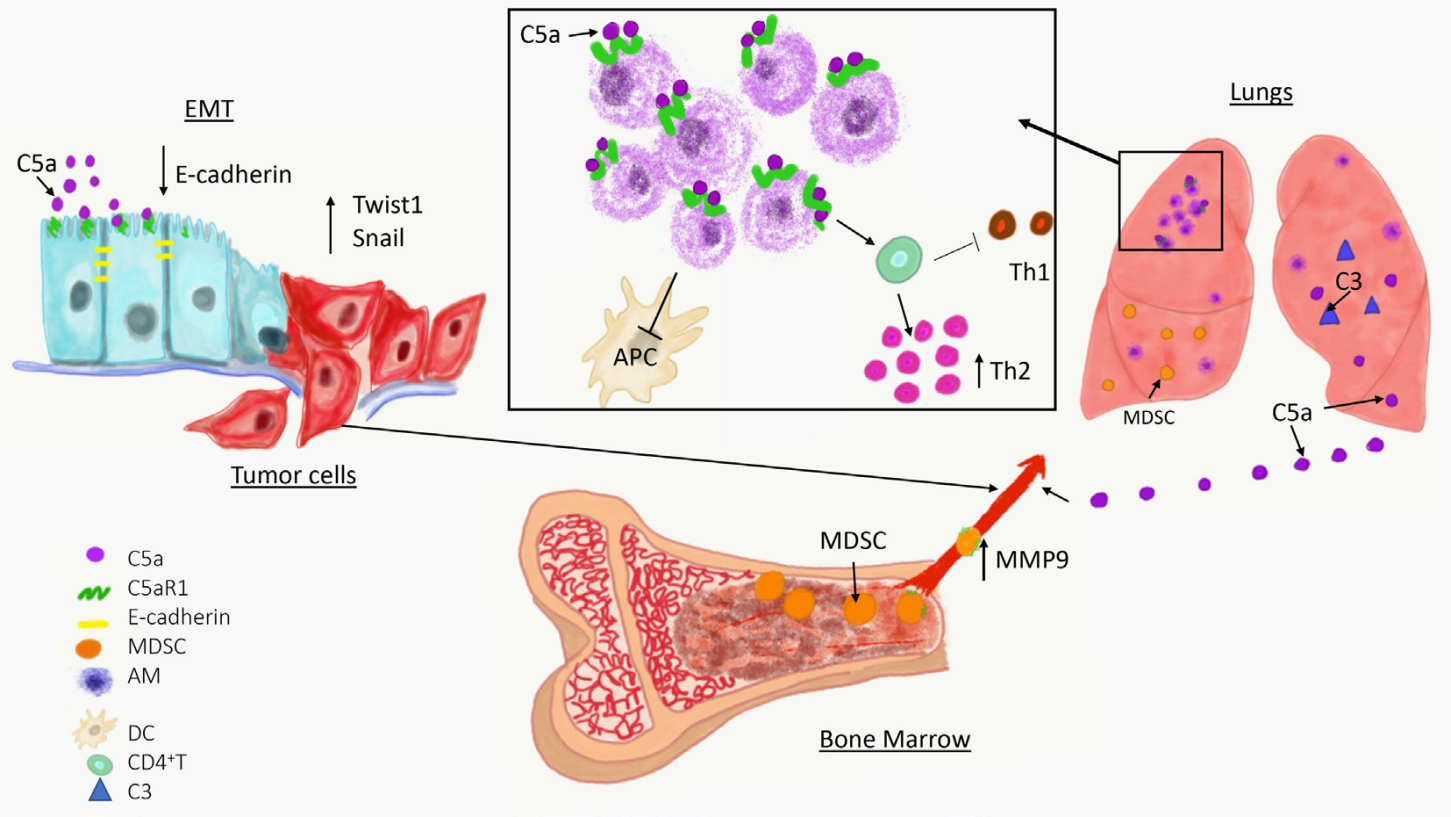
Overview of a role of complement in cancer metastasis. The C5a/C5aR1 axis contributes to the initial step of the invasion-metastasis cascade epithelial-to-mesenchymal transition, which is essential for tumor cell motility, invasion of extracellular matrix blood vessels. C5aR1 signaling contributes to the formation of the premetastatic niche by recruiting immunosuppressive myeloid-derived suppressor cell from bone marrow to the lungs and by regulating self-renewal of alveolar macrophages that impair antitumor immunity *via* reducing antigen-presenting capacity of dendritic cells (APC) and polarizing T cell response toward Th2 phenotype.

Complement has been linked to EMT in two recent studies (Figure 1) (60, 61). In the first study, increased expression of C5aR1 was found in hepatocellular carcinoma and hepatocellular carcinoma-derived cell lines and positively correlated with stage and invasion of liver capsule by tumor cells. The stimulation of C5aR1 *via* C5a induced EMT, as demonstrated by downregulation of E-cadherin and Claudin-1 expression, and upregulation of Snail. Mechanistically, C5aR1-mediated EMT was linked to ERK1/2 signaling (61). In another study, C3 expressed in ovarian carcinoma-derived cells reduced expression of E-cadherin through C3a and Krüppel-like factor 5. Interestingly, C3 expression in tumor cells is transcriptionally regulated by twist basic helix–loop–helix transcription factor 1 (TWIST1), which binds to the *C3* promoter and enhances its expression. TWIST1 and C3 colocalized at the invasive tumor edges, and in the neural crest and limb buds of mouse embryos. Therefore, this work identified TWIST1 as a transcription factor that regulates *C3* expression during pathologic and physiologic EMT (60). The phenotypes associated with EMT program resemble phenotypes of cancer stem cells (CSCs) that are essential for metastatic spread. The recent work showed that CD10^+^ cancer-associated fibroblasts that express a second C5a receptor (C5L2) provide a survival niche for CSCs through C5L2-mediated NF-kβ activation (62). Through EMT, tumor cells reduce their attachment to neighboring tumor cells and surrounding stromal elements, increase motility, and acquire the ability to invade stroma, blood, or lymphatic vessels, thereby gaining access to the vasculature.

The invasion of blood or lymphatic vessels enables tumor cells to intravasate and enter the circulation. The histopathological identification of vasculature invasion is itself a poor prognostic factor and often correlates with advanced metastatic disease (63). The lymph node metastases are a critical factor in cancer staging and are independent prognostic factors in several malignancies (64). However, mortality in cancer patients results from hematogenous spread to the vital organs including lungs, liver, and ultimately brain. Although initially lymph node metastases were thought to precede the subsequent hematogenous spread of cancer, evidence that draining lymph nodes are just temporary “parking” sites for cancer cells, before their departure to blood, is rather limited. It seems that lymph nodes represent a final destination for some cancer cells while other tumor cells, for unclear reasons, spread through the blood vessels (59). Upon successful intravasation, tumor cells move with the bloodstream to distant sites. However, only a small fraction of tumor cells that enter circulation safely reach their destination in the capillary beds of lungs and liver or cross the blood–brain barrier. This low efficacy of metastatic spread in blood results from hemodynamic stress and elimination of circulating tumor cells by the innate immunity, mainly natural killer (NK) cells (65). In contrast to NK cells, interactions with platelets (66) and neutrophils (67, 68) appear to facilitate metastasis.

After reaching their final destination, tumor cells are trapped in the capillary beds of the vital organs because their size is usually larger than the diameter of a single capillary. The halting of tumor cells in narrow capillaries facilitates their interaction with endothelium that is required for adhesion to endothelial cells and subsequent crossing of this endothelial barrier by extravasating tumor cells (transendothelial migration). Several substances secreted by tumor and host cells in the capillary beds enhance adhesiveness of tumor and endothelial cells and increase vascular permeability (69, 70), thereby, facilitating tumor cell extravasation. In the liver and kidneys, the fenestrated endothelium seems to facilitate seeding of these organs by metastasizing tumor cells. Perhaps the mechanisms contributing to extravasation of tumor cells in different organs vary, depending on the location and intrinsic properties of metastasizing cells.

After successful seeding of distant sites, tumor cells usually persist in an indolent state as single disseminated tumor cells or subclinical microscopic metastases, sometimes for years. The reasons for tumor cells to remain in a dormant state are unclear; however, poor adaptation of tumor cells to new microenvironment of metastasis-targeted organs seems to play a significant role (59). In addition, transition to rapidly growing and clinically overt metastasis, known as metastatic colonization, requires robust angiogenesis and immune evasion that may not be evident during a dormant phase of metastatic progression (71). For breast, prostate, and kidney cancers, a dormant phase may last even for decades after initial therapy and eradication of a primary tumor (59). Therefore, dormant tumor cells need to find a microenvironment-niche that allows them to slowly self-renew, provides needed nutrients, and protects from anticancer drugs and elimination by the immune system (72). For example, prostate carcinoma cells often metastasize to bones where they compete for residence in the endosteal niche with hematopoietic stem cells (73). In multiple organs including lungs, bones, and brain, tumor cells reside in close proximity to blood vessels in a region known as the perivascular niche (72, 74).

Interestingly, as much as EMT is necessary to trigger the invasion-metastasis cascade in primary sites, the reversal of this process, called mesenchymal-to-epithelial transition (MET), contributes to metastatic colonization, which is a final stage of metastatic disease. In metastatic tumors, MET appears to be critical in restoring a complex and heterogonous structure resembling primary tumors (75). Metastatic colonization leads to development of clinically overt and rapidly growing metastatic lesions, which are the ultimate reason for cancer-associated mortality. The transition from dormant to rapidly growing metastases requires acquisition of specific cellular programs by tumor cells, such as, discussed already MET, but also complex and well-orchestrated changes in the microenvironment of metastasis-targeted organs that include angiogenesis (72, 76), inflammation (77), remodeling of extracellular matrix (78, 79, 80), and evasion of antitumor immunity (81).

## The Premetastatic Niche

Surprisingly, in several mouse models of cancer, changes that appear to be essential for metastatic colonization, e.g., a final stage of the invasion-metastatic cascade, including vascular alterations, remodeling of extracellular matrix, inflammation, and immunosuppression are observed in certain organs that seem to be marked for metastasis even before the arrival of the tumor cells. These alterations, collectively known as the premetastatic niche, are thought to facilitate seeding of these organs by disseminated tumor cells and their survival after they arrive to distant sites. The establishment of the premetastatic niche is triggered by the primary tumors (82) because efficiency of seeding of metastasis-targeted organs by intravenously (i.v.) injected tumor cells is greatly enhanced by the presence of these tumors (3). Tumor-free mice i.v. injected with murine cancer cells developed significantly less lung metastases-derived from these i.v. injected cells than breast tumor-bearing mice i.v. injected with the same amounts of cells, indicating that the presence of primary breast malignancy facilitated seeding of the lungs by circulating (i.v. injected) tumor cells (3). It also appears that different types of cancer selectively prepare the premetastatic niche in different organs. This reflects the tendency of some malignancies to metastasize preferentially to specific locations. This specificity, known also as organotropism, was initially noted by Stephan Paget in 1889 (82), however, mechanisms regulating organotropism remain unclear until now. These mechanisms perhaps involve complex interactions between tumor cells and metastasis targeted organs that were proposed by Paget in his “seeds (tumor cells) and soil (microenvironment of premetastatic sites) theory.” However, until seminal studies of the last decade (83, 84), which indeed established the field of premetastatic niche, the experimental proof for Paget’s theory was missing. It is increasingly accepted that the premetastatic niche is created by tumor-secreted factors and tumor-shed extracellular vesicles, mainly exosomes. These factors seem to collectively control the stepwise development of premetastatic niche that begins with vascular alterations and progresses through activation of resident cells, extracellular matrix remodeling, and recruitment of bone marrow-derived cells (85).

### Secreted Factors

The evidence that tumor-secreted factors contribute to the premetastatic niche and organotropism, was, perhaps, first provided by experiments showing that melanoma-conditioned medium injected into mice, directed the metastasis of Lewis lung carcinoma cells (which normally metastasize only to the lungs) to sites typical for experimental melanoma metastasis (84). Among several identified factors secreted by tumors, vascular endothelial growth factor A (VEGFA), placental growth factor (84), transforming growth factor β (TGF-β), and tumor necrosis factor (TNF) were first demonstrated to prepare “soil” for tumor cells (26, 83).

### Exosomes

Exosomes, small extracellular vesicles formed on the cell surface through a budding mechanism, contain diverse cargo that facilitates cell-to-cell communication and homeostatic cell regulation (86). However, in patients and mouse models, formation of exosomes by tumor cells is increased compared to normal cells (87). Tumor-derived exosomes were isolated from plasma of cancer patients and mice with experimental tumors and found to carry tumor-derived cargo that promotes disease progression (87). This exosomal cargo, which includes tumor-derived miRNA and proteins, reprograms the target cells toward a pro-metastatic and pro-inflammatory phenotype, resulting in their contribution to the formation of the premetastatic niche. For example, melanoma B16-derived exosomes increase the expression of the receptor tyrosine kinase and MET in bone marrow progenitors, causing their exit from bone marrow and migration to the lungs, where they contributed to the premetastatic niche. Importantly, MET expression is also elevated in circulating CD45^−^C-KIT^low/+^TIE2^+^ bone marrow progenitors from patients with metastatic melanoma (87). B16-derived exosomes increase vascular permeability and enhance expression of TNF, S100A8, and S100A9, contributing to recruitment of bone marrow cells to the lung premetastatic niche. Of note, the source of S100 proteins was not identified in this study (87).

### Abundant Complement

In contrast to tumor-derived secreted factors and exosomes, complement proteins are present in abundance in plasma and body fluids (41, 88) and, therefore, are readily available to participate in the premetastatic niche in patients or mice even with very small tumors. Increased concentration of complement components in plasma and other bodily fluids has been observed in both cancer patients and mouse models of cancer (1, 2, 3) suggesting upregulation of the complement pathway. These higher amounts of complement proteins may be linked to enhanced expression and production of complement by the liver, however, local increases in expression of complement genes in tumors and organs targeted by metastasis contribute to augmented levels of complement fragments because endothelial and immune cells synthesize complement fragments (89, 90) and these cells are an integral component of the tumor microenvironment (81, 91) and the premetastatic niche (26). Thus, they are possible sites of origin for several complement proteins in tumors and metastatic sites. For example, in a mouse model of breast cancer with spontaneous metastatic spread mimicking human malignancy, increased concentrations of C3 were found in plasma and bronchoalveolar lavage indicating increased production of complement proteins (3). These higher levels of complement fragments correlated with increases in expression of C3 and C5 genes in the lungs (3, 92). Cytotoxic CD8^+^ T cells were found to synthesize C3 in mouse models of melanoma and breast cancer (51). Importantly, tumor cells also produce complement proteins. Mouse ovarian carcinoma tumor cells and human ovarian carcinoma cell lines were demonstrated to produce C3 (24). In a squamous cell carcinoma model, expression of C3, factor B, and factor I were also demonstrated (93, 94). Boire and colleagues recently demonstrated that C3 produced and secreted from tumor cells has prosurvival functions and facilitates leptomeningeal metastasis (95).

Complement proteins are secreted from cells in their inactive forms, as zymogens. To exert their functions, these fragments are activated through a series of proteolytic cleavages that form a complement cascade, which ends with generation of complement effectors (41). Therefore, if complement plays a role in regulating metastatic progression, complement activation in metastasis-targeted organs or tumors is anticipated. This activation can be revealed by detecting deposited complement fragments in tissue or secreted effectors such as complement anaphylatoxins, C3a, C4a, and C5a. The cleavage fragments of C3 were found to be deposited in the lungs prior to metastasis, indicating complement activation and participation of the complement system in the lung premetastatic (3). These data were obtained through a use of a syngeneic mouse model of metastatic breast cancer (4T1), in which tumor cells are injected into the mammary fat pad, and breast tumors formed there subsequently metastasize to distant sites, similar to human malignancy (96). The deposition of C3 cleavage fragments in the lungs correlated with increasing levels of C5a in plasma over time (Figure 1) (3).

### Vasculature

Increased vascular permeability is one of the earliest changes observed in the premetastatic niche and is associated with increased metastatic burden (97, 98). The factors secreted from primary tumors, including epithelial growth factor receptor ligand epiregulin, metalloproteinases MMP1, and MMP2, are known to impact vascular permeability in primary tumors and distant sites, helping tumor cells to intravasate in a primary and then extravasate in a distant site, respectively (99). In a melanoma model, tumor cells secrete factors upregulating angiopoietin 2, MMP3, and MMP10 that synergistically destabilize vasculature in the premetastatic organs (97). Factors affecting vascular permeability can also be secreted from different cell populations recruited to the premetastatic niche. For example, myeloid cells were shown to produce MMP9 (100) and VEGFA (101). Endothelial cells, which are often targets for vasoactive substances, can themselves participate in vascular alterations in the premetastatic niche. VEGFA-dependent upregulation of E-selectin on the luminal surface of endothelium facilities adhesion of tumor cells to endothelium and subsequent extravasation (102).

The complement effectors, especially C5a, are powerful inflammatory mediators that are actively engaged in bringing leukocytes to sites of inflammation. C5a can achieve this goal, acting as a potent chemoattractant that causes cytoskeleton changes in leukocytes that are responsible for cell movement (103). However, it also enhances (directly and indirectly) vascular permeability, further adding to accumulation of leukocytes in inflammatory foci (103). The complement C3 cleavage fragments were found to be deposited in the premetastatic lungs as early as 4 days after injecting tumor cells into the mammary fat pad in a model of breast cancer (before any tumor cells are present in the lungs). Since C3 is a central component of complement cascade, on which all complement activation pathways converge, deposition of C3 cleavage fragments indicates complement activation and subsequent generation of C5a (88), which indeed was present in sera of these mice (3). It is, therefore, conceivable that complement contributes directly to increased vascular permeability in the premetastatic niche similar to its participation in inflammatory vascular alterations; however, a direct experimental evidence for these C5a functions in the premetastatic niche has yet to be provided. The indirect impact of complement on vascular changes can be attributed to recruitment of MDSC (3) because these cells can produce and release several vasoactive factors including MMP9, which is intimately involved in regulating vascular integrity in the premetastatic niche (83, 84). Genetic ablation of *Mmp9* was shown to normalize the aberrant vasculature in the premetastatic lungs and reduce metastatic burden (100). The seminal recent work has also demonstrated that cancer-cell-derived C3/C3a through C3aR in the choroid plexus disrupts the blood–cerebrospinal fluid barrier. The increased permeability of this barrier facilities the entry of plasma proteins that are essential for tumor growth into the cerebrospinal fluid, thereby, facilitating leptomeningeal metastasis (95).

### Resident Cells

Resident cells in metastasis-targeted organs are naturally suited to participate in the premetastatic niche because they are present before the arrival of tumor cells. Tumors can reach and potentially hijack these cells through several mechanisms including secreted tumor-derived factors, exosomes, and recruitment of bone marrow-derived cells that subsequently interact with resident components of the premetastatic niche. Recent work also demonstrated that complement activation regulates resident cells in the lungs (Figure 1) (92). As discussed already, endothelial cells are targets for several vasoactive substances whether derived from tumors, recruited cells, or generated locally. Fibroblasts contribute to remodeling of extracellular matrix through deposition of new extracellular matrix components or by secreting enzymes that affect preexisting components of the matrix (104). S100A4 expressing pulmonary fibroblasts incorporate exosomal cargo derived from breast cancer cells and through this mechanism upregulates S100 proteins (82). Exosomal cargo from pancreatic carcinoma induces similar changes in the liver-resident macrophages, Kupffer cells (105). S100 proteins were linked to recruitment of myeloid-origin cells to the premetastatic niche (106). Similar to Kupffer cells in the liver, another population of tissue-resident macrophages, pulmonary alveolar macrophages, were recently demonstrated to contribute to the premetastatic niche (92).

These recent developments on participation of tissue-resident macrophages to metastasis are of particular interest because roles of these cells in cancer remain unclear, in contrast to very well-studied TAMs or inflammatory monocytes/macrophages recruited to the lungs with metastases by CCL2 (101). Several early studies yielded conflicting results on how liver Kupffer cells and lung alveolar macrophages contribute to cancer progression (107). A role of these cells in cancer requires revision because recently published linage-tracing data demonstrated that these cells have a different origin and biology than inflammatory macrophages (108). Unlike inflammatory macrophages and TAMs that are recruited from bone marrow to sites of inflammation or tumors, respectively, tissue-resident macrophages migrate to different organs during embryogenesis prior to hematopoiesis, and self-renew thereafter (109). Pulmonary alveolar macrophages are the resident macrophages of the lungs, and while they have well-known immunoregulatory and homeostatic roles in healthy lungs (110), they appear to be well-suited to partake in preconditioning the lungs for metastasis through their immunoregulatory properties. In support of this notion, alveolar macrophages were found to accumulate in premetastatic lungs and this accumulation was the result of cell proliferation rather than recruitment from bone marrow (92). The mechanisms controlling proliferation of these cells were linked to C5aR1 signaling because tumor-bearing C5aR1-deficient mice presented with a lower total number of these cells in the lungs compared to tumor-bearing wild-type controls and this reduced cell number associated with reduced Ki-67 expression. Immunoregulatory functions of these cells appeared to be related to skewing effector CD4^+^ T cells responses toward Th2 phenotype, which plays limited role in antitumor immunity in contrast to Th1 responses (92). In addition, alveolar macrophages in tumor-bearing hosts reduced number and antigen-presenting capacity of lung dendritic cells through regulation of TGF-β1 in lung infiltrating leukocytes (Figure 1). The depletion of alveolar macrophages reversed immunosuppression and reduced lung metastatic burden (92).

### Extracellular Matrix Remodeling

The continuous remodeling of extracellular matrix by tumor-derived secreted factors, resident fibroblasts, and recruited bone marrow-derived cells is an integral part of the premetastatic niche (111). This remodeling is achieved through deposition of new extracellular matrix components or modification of existing components. For example, deposition of fibronectin produced by activated fibroblast provides a docking site for bone marrow-derived cells that express fibronectin receptor VLA-4 (84). These stromal fibroblasts, stimulated with tumor-derived TGF-β, produce periostin in a mouse model of breast cancer (78). Periostin directly interacts with type I collagen, fibronectin, and Notch1 through its amino-terminal EMI domain and interacts with tenascin-C and BMP-1 through its fas I domains. These periostin interactions with mainly extracellular matrix molecules occur at first intracellularly. In addition, periostin serves as a ligand for integrins such as αvβ3 and αvβ5 and promotes cell motility by acting outside the cell (112). Periostin was demonstrated to facilitate melanoma metastasis to wounds (113) and to regulate immunosuppressive functions of MDSC during early stages of breast cancer metastasis (114). MDSC (mainly monocytic-MDSC), which accumulated in the premetastatic lung of MMTV-PyMT spontaneous breast tumor-bearing mice, secrete versican, an extracellular matrix proteoglycan. Versican contributed to MET and the formation of macrometastasis in the lungs (115). Enzymatic modulation of extracellular matrix proteins also occurs in the premetastatic niche and is mediated mainly by metalloproteinases produced by cells that are recruited to the premetastatic niche. In addition, the members of the LOX family crosslink collagen type I and IV and this crosslinking facilitates adhesion of bone marrow-derived cells to the extracellular matrix of the premetastatic niche. These cells produce more metalloproteinases contributing to further remodeling of extracellular matrix.

Although complement was not directly linked to extracellular-matrix remodeling in the premetastatic niche, studies in different model systems demonstrated that fibronectin can interact with several complement components including C1q (116) and C3 cleavage fragments (117). C3 cleavage fragments can also bind to different components of extracellular matrix including collagen (118). Interestingly, binding of C1q to fibronectin was not associated with complement activation but was connected to enhancement of phagocytosis of C1q coated particles through fibronectin. Therefore, functional significance of complement interactions with extracellular matrix proteins in the premetastatic niche remain to be elucidated. However, it is reasonable to theorize that complement C3 cleavage fragments, bound to extracellular matrix proteins, interact with its receptors broadly expressed on myeloid-origin cells that are recruited to the premetastatic niche. The receptors for C3 degradation products include CR1, which binds C3b and iC3b, CR2 (CD21), which binds the degradation products of C3b (iC3b, C3dg, C3d), CR3 (CD11b/CD18 or Mac-1), which binds iC3b, and CR4 (CD11c/CD18), which binds iC3b, however, through a different domain than CR3 (119). C5a leads to upregulation of CR3 on MDSC, which may facilitate adhesion of these cells to endothelium and recruitment to tumors (22); however, it may also contribute to adhesion of MDSC to extracellular matrix in the premetastatic niches.

### Recruited Cells

The identification of bone-marrow derived cells in the premetastatic niche and discovery of their roles in facilitating seeding of these niches by tumor cells, provided perhaps the first experimental evidence confirming the “seed and soil” theory (83, 84). A seminal work by Hiratsuka and colleagues defined Mac1 (CR3) positive macrophages as a source of MMP9 in the lungs in addition to endothelial cells. These macrophages were recruited to the lungs because resident alveolar macrophages do not express CD11b (https://www.immgen.org/). The study by Kaplan and colleagues demonstrated recruitment of VEGFR1 and VLA-4 expressing hematopoietic progenitors to the premetastatic lungs and their participation in the premetastatic niche (84). These studies opened an avenue for further investigations into discovery of other recruited components of the premetastatic niche. Less than a decade later, MDSC, which were long recognized as modulators of the primary tumor microenvironment (120), were identified as contributors to the premetastatic niche (100, 115). However, these studies reported on metastasis promoting properties of MDSC linked to increased vascular permeability (100) and remodeling of extracellular matrix (115) rather than to their well-established immunoregulatory roles.

The C5aR1/C5a signaling axis recruits MDSC to primary tumors (2, 22), therefore, it was explored whether similar mechanisms operate in the premetastatic niche. Utilizing a syngeneic mouse model of metastatic breast cancer, it has been demonstrated that C5aR1 knockout or wild-type mice administrated with a specific C5aR1 inhibitor (PMX-53) had decreased lung and liver metastatic burden compared to control mice. Interestingly, C5aR1 appear to regulate only metastasis in this model because lack of C5aR1in mice did not affect primary breast tumors. The differences in lung metastasis were associated with differences in a degree of infiltration of the lungs and livers by MDSC. The lung infiltrating MDSC were found mainly in interavleolar septa and due to intensity of this infiltration, the morphological picture resembled interstitial pneumonia. Therefore, the term the premetastatic pneumonia has been proposed to emphasize intensity of MDSC infiltration and specific localization of these cells in the lungs (3). These MDSC were recruited to the premetastatic sites through C5a since C5aR1 was expressed in blood MDSC and complement activation, leading to C5a generation, was observed in the premetastatic niche (Figure 1). To further investigate the role of C3 cleavage fragments and MDSC, tumor-draining lymph nodes from breast cancer patients were examined; they observed that C3 fragments’ deposition and local C3 production were both intensified in lymph nodes with metastases (3). The decreased metastasis in mice lacking C5aR1 resulted from improved antitumor immunity due to escalated infiltration of the lungs by CD8^+^ and CD4^+^ T cells. In addition, the increases in these T cell subsets were found in peripheral blood and C5aR1-deficiency favored Th1 response. Also observed was a decrease in Tregs in both the blood and the lungs in C5aR1-knockout mice. The T cell subsets including CD4^+^ and CD8^+^ T cells isolated from the lungs of C5aR1 knockout mice produced increased amounts of IFN-γ. The elimination of cytotoxic CD8^+^ T cells by neutralizing antibody erased the inhibitory effect of C5aR1-deficiency on metastasis, supporting notion that this effect was caused by stimulating antitumor immunity. Importantly, these data also indicate immunoregulatory functions of MDSC in the premetastatic niche (3).

## Concluding Remarks

The evidence supporting contributions of complement to cancer metastasis is scarce and limited to a few recent papers. However, it appears that complement affects key steps in the invasion-metastasis cascade including EMT and the premetastatic niche (Figure 1). Given ubiquitous presence of complement in body fluids and tissues, the potential contributions of complement to regulating metastasis are significant. Our recent work demonstrated that C5aR1 regulates resident (alveolar macrophages) (92) and recruited (MDSC) (3) cells in the premetastatic niche. The significance of this regulation was underscored by complete protection from lung metastasis in mice depleted of alveolar macrophages and treated with C5aR1 inhibitor (92). Thus, despite an early phase of studies on complement participation in metastasis, several complement fragments appear to be promising targets for therapies seeking to stop cancer metastasis.

## Author Contributions

All authors listed have made a substantial, direct, and intellectual contribution to the work, and approved it for publication.

## Conflict of Interest Statement

The authors declare that the research was conducted in the absence of any commercial or financial relationships that could be construed as a potential conflict of interest.
